# Molecular diversity of *Scutellonema bradys* populations from Benin, based on ITS1 rDNA and COI mtDNA

**DOI:** 10.1007/s40858-018-0221-5

**Published:** 2018-05-10

**Authors:** Sètondji Alban Paterne Etchiha Afoha, Antoine Affokpon, Lieven Waeyenberge, Nancy de Sutter, Clément Agbangla, Alexandre Dansi, Daniel L. Coyne, Nicole Viaene

**Affiliations:** 1 BIORAVE, Laboratory of Biotechnology, Genetic Resources and Animal and Plant Breeding/ UAC, University of Abomey-Calavi, Dassa, Benin; 2 FSA, Faculty of Agronomic sciences/ UAC, University of Abomey-Calavi, Abomey-Calavi, Benin; 3 ILVO, Flanders Research Institute for Agriculture, Fisheries and Food, 9820 Merelbeke, Belgium; 4 LGB, Laboratory of Genetic and Biotechnologies/ UAC, University of Abomey-Calavi, Abomey-Calavi, Benin; 5 IITA, IITA-Kenya, Kasarani, P.O. Box 30772-00100, Nairobi, Kenya; 6 UGENT, Department of Biology, Ghent University, K.L. Ledeganckstraat 35, 9000 Ghent, Belgium

**Keywords:** *Dioscorea* spp., Diagnostics, Phylogenetic analysis, Sequence comparison, Species-specific primer, Yam nematode

## Abstract

In Benin, yam production continues to face numerous production constraints, including yield and quality reduction by *Scutellonema bradys*. Implementation of efficient management techniques against this pest requires an improved understanding, including at the molecular level, of the pest. The current study aimed at identifying the *Scutellonema* spp. associated with yam in Benin and investigating the phylogenetic relationships between populations. Nematodes of the genus *Scutellonema* were obtained from tubers exhibiting external dry rot symptoms. DNA was extracted from nematodes belonging to 138 populations collected from 49 fields from 29 villages. For 51 of these populations, both the ITS1 and COI regions could be amplified *via* PCR, sequenced, compared with available sequences in the NCBI database and were identified as *S. bradys*. Maximum likelihood was used to construct 60% consensus phylogenetic trees based on 51 sequences. This phylogenetic analysis did not reveal any genetic separation between populations by cultivar, village, cropping system nor by agroecological zone. Neither could any subgroups within *S. bradys* be separated, indicating that no subspecies were present. An earlier published species-specific primer set was verified with the DNA of the 51 sequences and was considered a reliable and rapid method for *S. bradys* identification.

## Introduction

Yam (*Dioscorea* spp.) is a key starch staple crop contributing to food security and poverty alleviation in Benin (Loko et al. 2015). However, yam faces various constraints to production, especially plant-parasitic nematodes. These organisms induce significant tuber damage in terms of yield suppression and quality of produce (market value), negatively impacting on household income. The most harmful nematodes belong to the genera *Scutellonema* spp.*, Meloidogyne* spp. and *Pratylenchus* spp. (Bridge et al. 2005).

The genus *Scutellonema* contains several species, which are widely distributed across tropical and subtropical regions with more than 60% of the species recorded from Africa (Sher 1964; Siddiqi 2000). Besides the 45 valid species described by Siddiqi (2000), a few new species have been reported by several authors (Kolombia et al. 2017). These nematodes are primarily root ectoparasites, feeding from outside the roots and are associated with a large range of agricultural and horticultural crops (van den Berg et al. 2013). Three species, *S. bradys* (Steiner and LeHew) Andrássy, *S. cavenesi* (Sher) and *S. brachyurus* (Steiner) Andrássy, are considered agricultural pests. However, *S. bradys,* an important pest of yam, is unusual in that it feeds endoparasitically (Ayala and Acosta 1971; Bridge et al. 2005; Coyne et al. 2006a; Kolombia et al. 2017). It is reported as the most important nematode that affects yam in Benin (Baimey et al. 2009), responsible for dry rot of yam tubers, a degenerative disease which results in significant damage to field production and especially in postharvest losses during storage. Symptoms begin with cream to light yellow lesions developing below the skin of the tuber. They gradually enlarge and darken, sometimes turning dark brown or black. Surfaces may crack and skin parts flake off, exposing dark brown patches on infected tubers (Bridge et al. 2005). According to Coyne et al. (2012), the geographical center of origin for *S. bradys* is West Africa, in particular Benin and Nigeria. Recently, however, it was also reported infecting locally cultivated yam in East Africa (Coyne et al. 2016).

In West Africa, *S. bradys* was reported as a potential risk on potato (*Solanum tuberosum* L.), on which it can also cause substantial damage (Coyne et al. 2011; Mwamula et al. 2015). It has been found parasitising various crops from Africa, the Americas and Asia (Bridge et al. 2005). In the yam belt of West Africa, although the damage caused by *S. bradys* varied with the yam cultivar/species and agroecological zone (Baimey 2005; Coyne et al. 2006a) as well as with the population (Baimey et al. 2009), no difference in pathogenicity has been detected between *S. bradys* populations (Coyne et al. 2012). To date, no robust screening process on the wide diversity of yam cultivars in Benin has been undertaken.

Morphological identification of *Scutellonema* species is not simple because a number of species share very similar morphological characters (Kolombia et al. 2017). Phylogenetic and sequence analysis of rRNA and other genes were reported to provide attractive solutions to validate morphology-based identifications (van den Berg et al. 2013). Previous molecular studies, based on the ITS1–2 regions of the rDNA and PCR-RFLPs of 14 *S. bradys* populations collected throughout the West-African region, revealed a relatively high degree of genetic polymorphism both within and between populations (Coyne et al. 2006b). Genetic diversity was reported also by Kolombia et al. (2017) who studied the D2-D3 expansion segments of the 28 s rDNA and the COI sequence of the mtDNA of *Scutellonema* populations retrieved from yam fields in Nigeria and Ghana. Recently, *S. bradys*-specific primers have been developed and demonstrated to work with *S. bradys* populations from Costa Rica (Humphreys-Pereira et al. 2014). Its applicability for identification of Benin’s populations is unknown. This study reports on: i) the species identification of *Scutellonema* populations obtained from yam tubers collected from various localities in Benin, using ITS1 (rRNA) and COI (mtDNA) sequences; ii) the genetic relationships between Benin *S. bradys* populations using these sequences as phylogenetic markers; iii) the applicability of an available *S. bradys*-specific primer set on*S. bradys* populations from Benin.

## Materials and methods

### *Scutellonema* populations

**Fig. 1:**
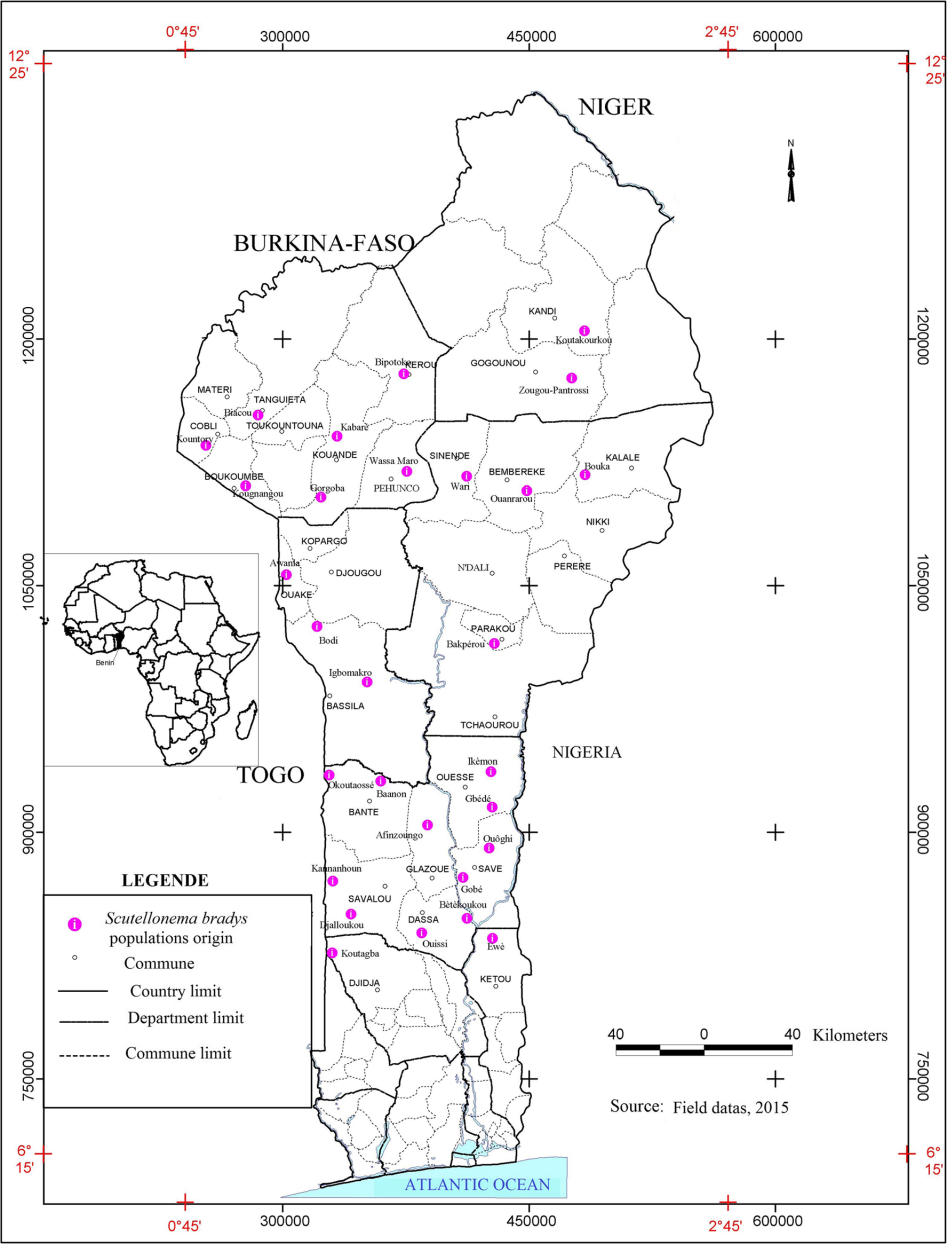
Map of Benin showing the villages where samples of yam tubers with symptoms of *Scutellonema* infection were taken

Populations of *Scutellonema* used in this study were obtained from yam tubers collected during the harvest period (December 2014 to February 2015) from 49 fields in 29 villages, distributed across four yam diversity zones in Benin (Fig. 1). According to Dansi et al. (1999), yam diversity zones in Benin are: (1) Bariba zone (North East) that includes Bariba cultural area; (2) Donga zone (North West) gathering the cultural areas Ani, Yom, Lokpa, Kotokoli; (3) Atacora zone (extreme North West) grouping the cultural areas Ditamari, Wama, Berba, Natimba, M’bermin; (4) Center-South zone with cultural areas Fè, Fon, Idatcha, Mahi and Tchabè. Each zone is characterized by different yam cultivars and cropping systems. One to three fields per village were sampled. Fields with different cultivars were selected and tubers with visual dry rot symptoms were selected. A total of 138 samples (populations) were collected.

For extraction of nematodes from tubers, the centrifugal flotation technique was used. Each tuber was first washed and then peeled using a kitchen vegetable peeler, before removing a 25 g subsample of peels (outer cortex) from the bulked peels (Coyne et al. 2006a; Affokpon et al. 2011). For samples analyzed in Benin, peels were macerated in a blender and the mixture passed through nested sieves (200 μm and 100 μm). The suspension, containing nematodes smaller than 100 μm, was then distributed into 50 ml test tubes and thoroughly mixed with kaolin. The amount of kaolin was 8% of the weight of the suspension, adjusted for each tube. The test tubes containing the suspension-kaolin mixture were subject to a first centrifugation at 3500 rpm for 7 min. After that, the supernatant was discarded and the deposit was mixed with sucrose (250 g/l) and centrifuged again at 3500 rpm for 4 min. The supernatant suspension (containing nematodes and sucrose) was collected on a 10-μm sieve and thoroughly rinsed with tap water to remove the sucrose. Peel subsamples sent to Belgium were analyzed according to the same procedure, except that the peel mixture was poured over an 850-μm sieve and centrifuging was performed with the automated zonal centrifuge (Hendrickx 1995) using MgSO_4_, instead of manually and using sucrose.

Nematodes were identified to genus level using a stereo microscope (Wild M5). For each sample, a maximum of 30 individuals of *Scutellonema* specimens were hand-picked and transferred into staining glasses containing 25 μl of milliQ water to rinse the nematode body. Specimens were then individually transferred into labeled eppendorf tubes containing 25 μl sterile water. Five tubes containing one individual nematode specimen and one tube containing 5 individuals were prepared for each of the 138 samples.

### DNA extraction

DNA extraction was performed following Holterman et al. (2006). An equal volume (25 μl) of Lysis Buffer (0.2 M NaCl, 0.2 M Tris-HCl pH 8.0, 1% beta-mercaptoethanol and 800 μg/ml proteinase-K), made shortly before DNA extraction, was added to each tube. Tubes were then incubated for 1.5 h at 65 °C followed by 5 min at 99 °C in a thermocycler. After incubation, tubes were stored at −20 °C.

### Amplification *via* polymerase chain reaction (PCR)

An amount of DNA recovered during the DNA extraction was amplified by the Polymerase Chain Reaction (PCR) (Mullis et al. 1986). Two different regions were examined. The first focused on the Internal Transcribed Spacer (ITS1) of the rDNA (ribosomal DNA) using VrainILVO18Sf (forward) and rRNASM5rev (reverse) primers (Vrain et al. 1992). Only samples for which the ITS1 region could be amplified were used for a second PCR, amplifying the cytochrome oxidase subunit I (COI) region of the mtDNA (mitochondrial DNA) using COI-JB3 (forward) and COI-JB5 (reverse) as primers (Derycke et al. 2010). PCR was carried out using a volume of 50 μl (1 μl of each crude DNA extract +49 μl of Master Mix). The Master Mix was prepared as following: 42 μl of sterile water; 5 μl of 10X Pfu Buffer with MgSO4; 1 μl of dNTPs (10 mM each); 0.3 μl of the forward and reverse primers (50 μM) mentioned above; 0.4 μl of Pfu DNA Polymerase (Fermentas). A DNA-polymerase with proof-reading activity was used to obtain a PCR-product with very low or no nucleotide incorporation errors. For some templates, the PCR-mix holding Pfu DNA polymerase was replaced by BIO-X-act short mix (Bioline) containing a ‘normal’ DNA polymerase. The BIO-X-act short mix is designed to work on problematic templates. Using this kit, the Master Mix was prepared as follows: 23.4 μl of sterile water; 25 μl of BIO-X-act short mix (Bioline); 0.3 μl of the same forward and reverse primers. The PCR program comprises an initial DNA denaturation during 3 min at 95 °C (Pfu) or 96 °C (BIO-X-act short mix), 35 cycles including DNA denaturation during 30 s at 95 °C (Pfu) or 96 °C (BIO-X-act short mix), primer annealing during 30 s at 60 °C (for ITS1 primer set) and 41 °C (for CO1 primer set), Extension during 2 min (Pfu) or 1 min (BIO-X-act short mix) at 72 °C and final extension during 10 min at 72 °C. After PCR, 5 μl of loading dye were added to each tube and 5 μl of each mixture was loaded on a 1.5% of agarose gel during 25 min at 100 V for electrophoresis. Then, the gel was stained with ethidium bromide (EtBr) solution (100 μl of EtBr in 1 l of water). After at least 10 min of soaking time, the gel was washed to remove excess of EtBr, visualized on a UV transilluminator and photographed.

### Reamplification

After electrophoresis, weak bands representing low yield of PCR-product, were reamplified using the band-stab method (Bjourson and Cooper 1992) to obtain a stronger band for purification. To do so, at least 10 μl of each PCR product was reloaded on 1.5% of agarose gel and stained as described above. Under UV light, each band was stabbed with a pipette tip and the tip was soaked in 49 μl of master mix. PCR followed by electrophoresis were repeated as described above to check if the bands were strong enough, representing good yield, for purification.

### PCR products purification, quantification and sequencing

The PCR products of the samples which showed strong bands for ITS1 and COI were purified using the Promega kit Wizard® SV Gel and PCR Clean-Up System. After purification, an amount of 1.5 μl of each purified PCR product was used to check the nucleic acid concentration using a spectrophotometer (Nanodrop). For the samples with sufficient concentration (minimum of 15 ng/μl), purified PCR product (5 μl) were mixed separately with 5 μl of 5 μM forward or reverse primer solution to sequence both strands of the PCR-product. Each total of 10 μl was sent for sequencing (Macrogen). The results were compared with sequences available in the National Center of Biotechnology Information (NCBI, USA) database for identification.

### Phylogenetic analysis

All sequences were analysed using the software program BioNumerics 7.5 (Applied Maths). This program automatically combines both sequences of the same sample (forward and reverse sequence) into one sequence called a contig. In cases when a contig could not be made due to the lack of an overlap between both sequences, the contig was created manually using the programs Chromas Lite 2.1 (Technelysium, Australia) and BioEdit version 7.1.3.0 (Hall 1999). The sequences of the samples where both regions (ITS1 and COI) could be amplified successfully were loaded into the Mega 6 software program (Tamura et al. 2013) to create a phylogenetic tree (Maximum Likelihood) to visualize possible subgroups within the species *S. bradys*. In addition, GenBank’s ITS1and COI sequences of *S. bradys* (7 sequences for ITS1 and 1 for COI) and*S. brachyurus* (3 sequences for each of ITS1 and COI) were added as references. During the phylogenetic analysis the topography of the trees were tested by bootstrapping (500 repeats). Finally, a 60% consensus tree was constructed.

### Assessing the efficiency of *Scutellonema bradys*-specific primers

The DNA of the samples for which sequences revealed the presence of*S. bradys* was used for PCR using the forward and reverse *S. bradys*-specific primers, SBVF1 (5′- CCTCTCCATGTGTCCCACTT-3′) and SBLR (5’TGCACAAGGCACACATCT-3′) developed by Humphreys-Pereira et al. (2014). PCR was carried out in a volume of 25 μl comprising 2 μl of DNA with 23 μl of Master Mix prepared following the Fermentas protocol: 19.5 μl of water; 2.5 μl of 10X Pfu buffer with MgSO4; 0.5 μl of dNTPs; 0.15 μl of the forward and reverse primers as mentioned above; 0.2 μl of Pfu DNA polymerase. Amplification conditions were as described by Humphreys-Pereira et al. (2014): 94 °C for 5 min, then 35 cycles of 95 °C for 30 s, 60 °C for 30 s and 72 °C for 60 s. The final extension was at 72 °C for 7 min. PCR products were separated by electrophoresis and stained as for analysis of the ITS or COI region.

## Results

### Sequences comparison of ITS1 and COI regions and phylogenetic analysis

**Fig. 2:**
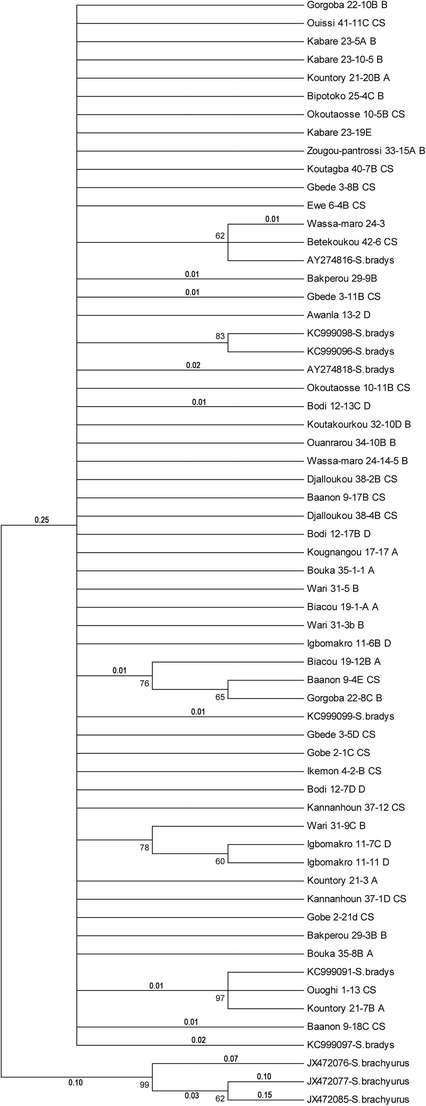
Phylogenetic 60% consensus ML-tree of the ITS1-based sequences of 51 populations of *S. bradys* from Benin (see Table 1 for code) and 10 reference sequences from GenBank. Numbers between 60 and 100 are bootstrap values and those smaller than 1 are the distance values which were representative for the number of substitutions. Distance values lower than 0.01 are not mentioned

Not all 138 samples produced sequences with sufficient quality for analysis, although *Scutellonema* sp. were obtained from all samples. Finally, a total of 51 sequences (Table 1) for both ITS1 and COI derived from the same samples were adequate for phylogenetic analysis and were compared with sequences available in the NCBI database. All were identified as belonging to *S. bradys*. The sizes of our sequences varied among samples and can be consulted in the NCBI databank where all sequences have been deposited (Table 1). A 60% consensus tree was generated. In the ITS1-based ML-tree (Maximum Likelihood) (Fig. 2), two separate groups were visible. The first including all of the 51 sequences and seven *S. bradys* sequences from GenBank (AY274816-*S.bradys*, KC999098-*S.bradys*, KC999096-*S.bradys,* AY274818-*S.bradys*, KC999099-*S.bradys*, KC999091-*S.bradys*, KC999097-*S.bradys*). In this group and with decreasing consensus percentage, smaller subgroups were visible but without any specificity in terms of origin: yam host, village, diversity or agroecological zone. The three sequences of *S. brachyurus* (JX472076-*S.brachyurus*, JX472077-*S.brachyurus* and JX472085*-S.brachyurus*) were gathered in the second group.

**Table 1:** Origins and codes of 51 *Scutellonema bradys* populations of which sequences were used for phylogenetic analysis, including their GenBank accession numbers

Villages	FN^a^	Cultivars	Codes	DZ^b^	AEZ^c^	GenBank	
						ITS1	CO1
Koutakourkou	38	Kpan anyé	32–10 B	B	SZ	MG938395	MG973136
Zougou-pantrossi	39	Kourôkagouroko	33-15A B	B	SZ	MG938396	MG973137
Biacou	20	Paanonnan	19-1A A	A	SZ	MG938377	MG973118
	21	Unspecified	19-12B A	A	SZ	MG938378	MG973119
Bipotoko	32	Sankourou pika	25–4 B	B	SZ	MG938389	MG973130
Gorgoba	26	Aïssoradjè danindjè	22–10 B	B	GZ	MG938383	MG973124
	25	Sakatadjè danindjè	22-8C B	B	GZ	MG938382	MG973123
Kabaré	27	Môrôkôrou	23–5 B	B	GZ	MG938384	MG973125
	28	Môrôkôrou	23–10-5 B	B	GZ	MG938385	MG973126
	29	Yombini	23-19E B	B	GZ	MG938386	MG973127
Kougnangou	19	PDRT	17–17 A	A	GZ	MG938376	MG973117
Kountory	23	Bénbéntingou	21-7B A	A	GZ	MG938380	MG973121
	24	Bénbéntingou	21–20B A	A	GZ	MG938381	MG973122
	22	Ounonyahou	21–3 A	A	GZ	MG938379	MG973120
Wassa maro	31	Baniouré pika	24–14 B	B	GZ	MG938388	MG973129
	30	Sankourou bakarou	24–3 B	B	GZ	MG938387	MG973128
Bakpérou	34	Gooroko	29–9 B	B	GZ	MG938391	MG973132
	33	Sankourou Souanrou	29–3 B	B	GZ	MG938390	MG973131
Bouka	41	Kokorogbanou	35–1 B	B	GZ	MG938398	MG973139
	42	Soussou	35–8 B	B	GZ	MG938399	MG973140
Ouanrarou	40	Terkokorou	34–10 B	B	GZ	MG938397	MG973138
Wari	35	Baniouré yintéguérou	31–3 B	B	GZ	MG938392	MG973133
	36	Douroubaéssirou	31–5 B	B	GZ	MG938393	MG973134
	37	Sankourou kpikourou	31–9 B	B	GZ	MG938394	MG973135
Baanon	11	Akpata	9–18 CS	CS	SHSZ	MG938366	MG973107
	10	Oboti	9–17 CS	CS	SHSZ	MG938365	MG973106
	9	Sôgôdôï	9–4 E CS	CS	SHSZ	MG938364	MG973102
Bètèkoukou	49	Môrôkô	42–6 CS	CS	SHSZ	MG938406	MG973147
Djalloukou	45	Sôgôdôï	38-2B CS	CS	SHSZ	MG938402	MG973143
	46	Tchakatchaka	38–4 CS	CS	SHSZ	MG938403	MG973144
Gbédé	5	Giladja	3–11 CS	CS	SHSZ	MG938361	MG973103
	5	Kokouman	3-8b CS	CS	SHSZ	MG938360	MG973101
	4	Vassarou	3-5D CS	CS	SHSZ	MG938359	MG973100
Gobé	3	Kablètona	2–21 CS	CS	SHSZ	MG938358	MG973099
	2	Kokoro	2–1 CS	CS	SHSZ	MG938357	MG973098
Ikèmon	6	Egniffoun	4-2B CS	CS	SHSZ	MG938362	MG973104
Kannanhoun	43	Mondji	37–1 CS	CS	SHSZ	MG938400	MG973141
	44	Sétéboua	37–12 CS	CS	SHSZ	MG938401	MG973142
Okoutaossé	12	Réguédé	10–5B CS	CS	SHSZ	MG938367	MG973108
	13	Réguédé	10-11B CS	CS	SHSZ	MG938368	MG973109
Ouissi	48	Kokoro Ika awodi	41-11C CS	CS	SHSZ	MG938405	MG973146
Ouôghi	1	Inanwaï	1–13 CS	CS	SHSZ	MG938356	MG973097
Awanla	18	Tchaabim	13–2-b D	D	GZ	MG938375	MG973116
Bodi	16	Kokoro Korokori	12–7 D	D	GZ	MG938372	MG973113
	17	Ourtani	12-17B D	D	GZ	MG938373	MG973115
	17	Sintêrê	12-13C D	D	GZ	MG938373	MG973114
Igbomakro	15	Agatou	11–11 D	D	GZ	MG938371	MG973112
	14	Kaki	11–7 D	D	GZ	MG938370	MG973111
	14	Kpadjiba kôkpô	11–6 D	D	GZ	MG938369	MG973110
Ewè	7	Wonni ibou	6–4 CS	CS	HFZ	MG938363	MG973105
Koutagba	47	Kpinnin (Labôkô)	40–7 CS	CS	SHSZ	MG938404	MG973145

^a^Field number;

^b^Diversity zones; B=Bariba; A = Atacora; D = Donga; CS = Center-South;

^c^Agroecological zones; SZ = Soudanian Zone; SHSZ = Sub-Humid Savannah Zone; GZ = Guinean Zone; HFZ = Humid Forest Zone

**Fig. 3:**
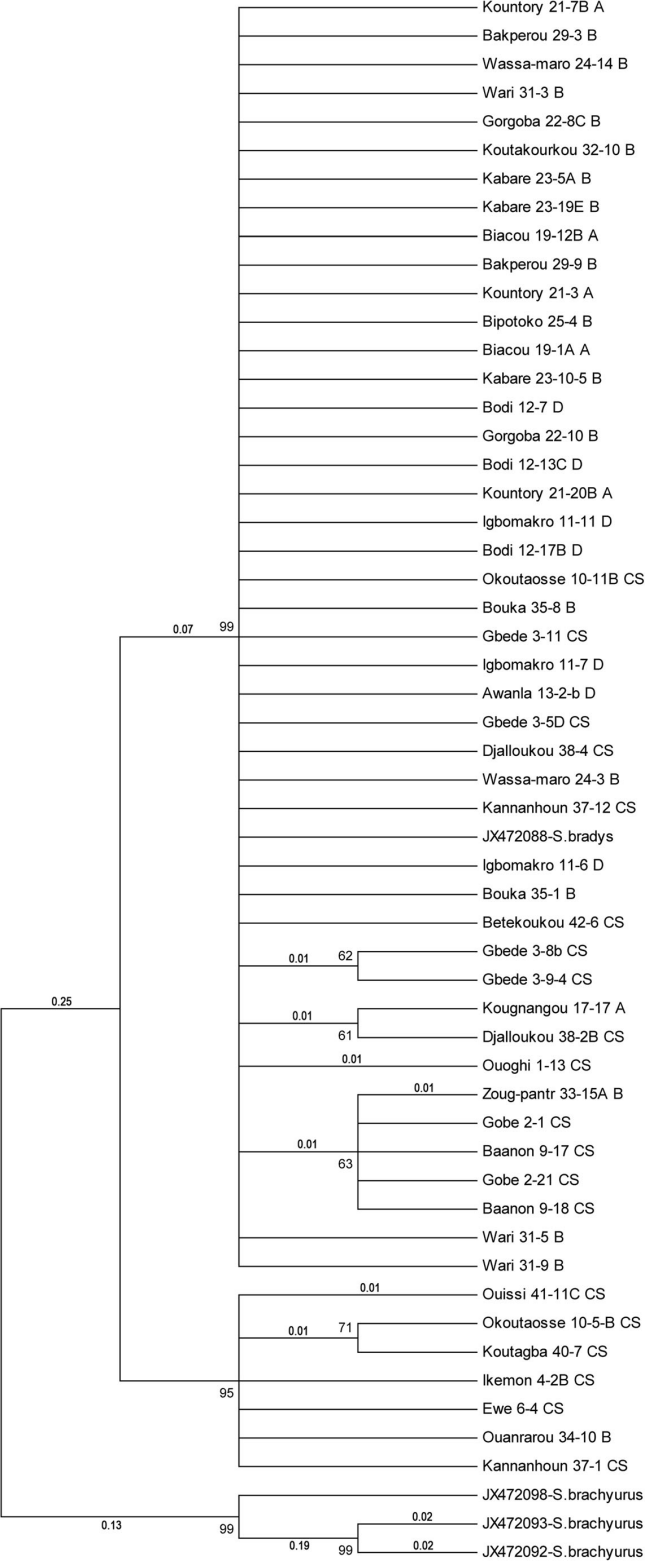
Phylogenetic 60% consensus ML-tree of the COI-based sequences of 51 populations of *S. bradys* from Benin (see Table 1 for code) and four reference sequences from GenBank. Numbers between 60 and 100 are bootstrap values and those smaller than 1 are the distance values which were representative for the number of substitutions. Distance values lower than 0.01 are not mentioned

In the COI-based ML-tree (Maximum Likelihood) (Fig. 3), three separate groups were visible. The first comprised 44 sequences and one *S. bradys* sequence from GenBank (JX472088-*S.bradys*). These 44 sequences belong to *S. bradys* extracted from different cultivars collected from different villages belonging to different agroecological and diversity zones. Three subgroups were visible in this group, with decreasing consensus percentages, but without practical meaning or specific properties. The second group assembled seven sequences of which six belong to the same agroecological (sub-humid Savannah) and diversity (Center-South) zones. The third group arranged the sequences of *S. brachyurus* (JX472098-*S.brachyurus*, JX472093-*S.brachyurus* and JX472092*-S.brachyurus*).

The subgroups in the COI-based ML-tree are not visible in the ITS-based ML-tree, and *vice versa*, small subgroups in the ITS-based ML-tree are not visible in the COI ML-tree.

### *Scutellonema bradys*-specific primers efficiency assessment

**Fig. 4:**
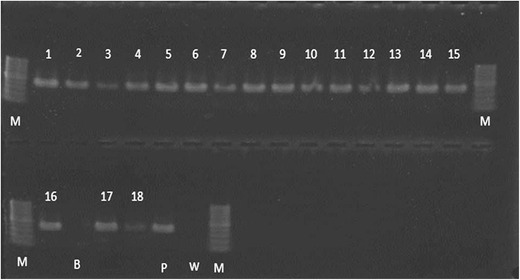
Results of the species-specific PCR for *S. bradys* with 18 samples (lanes 1–18). *M = 100 bp DNA marker (Promega), B=Blank lane, P = Positive control (S. bradys); W=Water*

All the 51 samples positive for *S. bradys* based on sequence analysis were also positive with the species-specific primers (Fig. 4).

## Discussion

Of the 138 nematode samples, only 51 (36.95%) yielded sequences of sufficient quality. Probably deterioration of the nematodes during transportation of nematode suspensions to Belgium led to problems with DNA extraction, yielding insufficient amounts of DNA. It is known that the efficiency of DNA extraction from a sample depends on the quality of nematodes (Perry et al. 2007). There was a difference between the quality of DNA extraction from nematodes extracted in Benin and transported in water suspension to Belgium and DNA extraction from nematodes freshly extracted from peels. This indicates that nematodes freshly extracted from peels are more efficient for molecular diagnostics than those in a water suspension. Perry et al. (2007) reported that if the period between nematode extraction and molecular analyses is several days or weeks, nematodes should be kept at low temperatures before use. According to the authors, the best approach is to use live nematodes or to kill them by heating briefly but leave the DNA undamaged for diagnostics. During extended field sampling visits, fixation in 75–90% alcohol, glycerol or simply drying the nematodes in a plastic tube are alternative methods to preserve nematode DNA for further molecular study.

The 51 populations for which it was possible to study the ITS1 and COI regions can be considered as representative for the yam growing regions of Benin. They originated from 28 villages and each of the four agroecological and diversity zones.

Sequence comparison of ITS1 and COI regions and phylogenetic analysis enabled a distinctive separation between the *S. brachyurus* sequences and the group with the sequences of *S. bradys*. This indicates that *S. bradys* populations from Benin are monophyletic and clearly distinct from other *Scutellonema* species. Within the *S. bradys* sequences, no genetic separation was visible, neither by yam cultivar from which nematodes were extracted nor by the corresponding villages, their diversity zone or the agroecological zone. This can possibly be explained by the extensive exchange of planting material between yam farmers. Yam exchange flow, including planting material, between producers in Benin has been reported previously (Loko et al. 2013; Baco et al. 2014). Dissemination of nematodes can occur from one region to another, when a population of *S. bradys* originating from one place can be present in low densities in an apparently healthy tuber. Comparatively low densities of nematodes occurring in tubers without any external symptoms of damage have been reported in Nigeria (Bridge 1973; Kolombia et al. 2017). Similar results were obtained with*S. bradys* populations in Costa Rica, where no separation by locality or by yam host were noticed (Humphreys-Pereira et al. 2014). Our finding corroborates those of Coyne et al. (2006a) and Bridge et al. (2005), who reported that, as yam is a vegetative propagated crop, untreated infected material used for planting perpetuates the disease cycle.

Subgroups observed with both ITS1 and COI DNA regions can be attributed to the molecular polymorphism and the genetic diversity within *S. bradys* populations. However, they are few in number and not well supported phylogenetically. In this way, our results do not support partly the conclusions of Coyne et al. (2006b, 2012) and Kolombia et al. (2017) who reported the relatively high degree of polymorphism both within and between West African*S. bradys* populations, indicating genetic diversity within as well as between populations. As *S. bradys* was spread rapidly through intensive exchange of planting material and the polymorphisms cannot be linked to any biological factor, there is no reason to consider them as separate subspecies.

Maximum likelihood (ML) is generally considered to make the most efficient use of data to provide the most accurate estimates of phylogeny. The basic idea is to compute the probability of the observed data assuming it has evolved under a particular evolutionary tree and a given probabilistic model of substitution (Subbotin et al. 2013). Both the 60% consensus COI-based ML-tree and the ITS1-based ML-tree show that the visible subgroups were not very well supported due to the lower bootstrap value (less than 70). Also, the visible subgroups in the COI-based ML-tree were not confirmed in the ITS-based ML-tree. Similarly, the smaller subgroups in the ITS-based ML-tree were not visible in the COI ML-tree. This supports our conclusion that there is no indication of subspecies in the *S. bradys* populations in Benin. Consequently, one given population can be used to screen all yam cultivars for resistance to *S. bradys*. Moreover, Coyne et al. (2012) reported that greater variability of *S. bradys* damage may occur because of environmental effects and host differences, than due to differences in pathogenicity of these populations. Therefore, on-farm screening in the different agroecological zones where different environmental conditions prevail is preferred over screening with different populations at one location. This is the first extensive molecular phylogenetic analysis of *S. bradys* populations from Benin.

In addition, the current study confirms that the species-specific primer set described by Humphreys-Pereira et al. (2014) is a reliable and rapid method for *S. bradys* identification. This primer set could be used for high throughput analysis of samples from yam and yam fields to detect *S. bradys*.
